# Ligation of left gastric vein may cause delayed gastric emptying after pancreatoduodenectomy: a retrospective study

**DOI:** 10.1186/s12876-022-02478-5

**Published:** 2022-08-26

**Authors:** Koichi Kimura, Ryosuke Minagawa, Takuma Izumi, Akihiko Otake, Takehiko Aoyagi, Daisuke Taniguchi, Hiroko Yano, Yuichiro Kajiwara, Kazuhito Minami, Takashi Nishizaki

**Affiliations:** Department of Surgery, Matsuyama Red Cross Hospital, 1, Bunkyomachi, Matsuyama City, Ehime 790-8524 Japan

**Keywords:** Obstructive jaundice, Pancreatic fistula, Risk factor, Sepsis, Stomach

## Abstract

**Background:**

This study aimed to determine which running pattern of the left gastric vein (LGV) is most frequently ligated in subtotal stomach-preserving pancreatoduodenectomy (SSPPD) and how LGV ligation affects delayed gastric emptying (DGE) after SSPPD.

**Methods:**

We retrospectively analysed 105 patients who underwent SSPPD between January 2016 and September 2021. We classified the running pattern of LGV as follows: type 1 runs dorsal to the common hepatic artery (CHA) or splenic artery (SpA) to join the portal vein (PV), type 2 runs dorsal to the CHA or SpA and joins the splenic vein, type 3 runs ventral to the CHA or SpA and joins the PV, and type 4 runs ventral to the CHA or SpA and joins the SpV. Univariate and multivariate analyses were used to identify differences between patients with and without DGE after SSPPD.

**Results:**

Type 1 LGV running pattern was observed in 47 cases (44.8%), type 2 in 23 (21.9%), type 3 in 12 (11.4%), and type 4 in 23 (21.9%). The ligation rate was significantly higher in type 3 (75.0%) LGVs (*p* < 0.0001). Preoperative obstructive jaundice (*p* = 0.0306), LGV ligation (*p* < 0.0001), grade B or C pancreatic fistula (*p* = 0.0116), and sepsis (*p* = 0.0123) were risk factors for DGE in the univariate analysis. Multivariate analysis showed that LGV ligation was an independent risk factor for DGE (odds ratio: 13.60, 95% confidence interval: 3.80–48.68, *p* < 0.0001).

**Conclusion:**

Type 3 LGVs are often ligated because they impede lymph node dissection; however, LGV preservation may reduce the occurrence of DGE after SSPPD.

**Supplementary Information:**

The online version contains supplementary material available at 10.1186/s12876-022-02478-5.

## Background

Pancreatoduodenectomy (PD) is the standard surgery for tumours in the pancreatic head, distal common bile duct, and duodenal papilla [1]. Despite the recent establishment and improvement of various operative techniques and perioperative management protocols, PD still results in several postoperative complications [2, 3].

Delayed gastric emptying (DGE) is a complication of PD, occurring in 19–57% of cases [4]. Although DGE is not life-threatening, it exacerbates patients' quality of life and prolongs hospitalisation after surgery [5]. Surgeons have cogitated how to prevent the development of DGE after PD. Several studies have reported that pylorus-preserving PD (PPPD) [6], ischaemic anastomotic sites of alimentary tract reconstruction, and injury of the branches of the vagus nerve [4] could be risk factors for the development of DGE after PD. Moreover, intra-abdominal infection and postoperative pancreatic fistula (POPF) are well-known risk factors for DGE after PD [7]. In addition, the retrocolic route for alimentary tract reconstruction [1], absence of Braun enteroenterostomy [8], and Roux-en-Y reconstruction method [9] have been reported as risk factors for DGE after PD.

However, little attention has been focused on gastric venous congestion following ligation of the coronary vein, which consists of the LGV and right gastric vein. Surgeons often encounter situations where the LGV requires ligation during lymph node dissection around the PV and/or CHA. The LGV occasionally obstructs the surgical field of vision in lymph node dissection and can be injured and cause bleeding or impede the surgery through other factors. Kurosaki et al. reported that preservation of the LGV reduces the occurrence of DGE after PPPD [10]. They suggested that LGV ligation induces antroduodenal congestion after PPPD. However, the effect of LGV ligation on DGE after subtotal stomach-preserving pancreatoduodenectomy (SSPPD), in which the pyloric ring is excised, remains unclear.

In this study, we classified the original running pattern of the LGV and investigated which type of LGV was most frequently ligated. Furthermore, we examined the effect of LGV ligation on DGE after SSPPDs.

## Methods

In this study, we retrospectively investigated the running pattern of the LGV and development of DGE after ligation of the LGV in SSPPD.

### Patients

A total of 105 adults (> 20 years old) who underwent SSPPD for pancreatic head tumours, periampullary tumours, or for diseases manifesting as a pancreatic mass at Matsuyama Red Cross Hospital between January 2016 and September 2021 were included in this study. Patients scheduled to undergo total pancreatectomy, combined liver resection, and pancreaticogastrostomy and those with a history of gastric surgery or colorectal surgery were excluded. All treatment procedures were performed after informed consent was obtained from the patients. Medical charts were retrospectively reviewed to obtain patient data. This study was approved by the Clinical Study Examination Committee of the Matsuyama Red Cross Hospital (Approval No. 919) and was performed in accordance with the ethical standards of the 1964 Declaration of Helsinki and its subsequent amendments.

### Surgical procedures

SSPPD with lymph node resection was performed as a standard procedure for pancreatic head, distal common bile duct, or periampullary tumours. The SSPPD involved a division of the stomach 3 cm proximal to the pyloric ring, followed by resection of the entire duodenum, gallbladder, distal common bile duct, and pancreatic head.

We resected the right gastric artery and vein for routine dissection of #5 lymph node for all surgical procedures in SSPPD [11]. Simultaneously, we performed #12 and #8 lymph node dissections and resection of the gastroduodenal artery. The inferior pancreaticoduodenal artery was resected routinely. The lymph nodes of the right semicircle of the superior mesenteric artery were dissected (#14 lymph node dissection) for pancreatic carcinomas.

Reconstruction was performed using the modified Child method. Either Roux-en-Y or Billroth II reconstruction was performed using the modified Child method. Pancreaticojejunostomy was performed using the modified Blumgart anastomosis [12] or Kakita method [13]. There were no restrictions on the hepaticojejunostomy technique used. Alimentary tract reconstruction as side-to-side gastroenterostomy was performed by using an automatic stapling device or hand-sewn anastomosis. Braun anastomosis was performed in all patients with Billroth II reconstruction.

Antecolic alimentary tract reconstruction was performed in all cases. During antecolic reconstruction, the anastomosis was positioned anterior to the transverse colon. The number, types, and locations of the intra-abdominal drainage tubes were determined according to the surgeon's preference.

### Postoperative management

The patients were administered epidural anaesthesia for 3 days, as appropriate. Early mobilisation was encouraged. The nasogastric tube (NGT) was routinely removed on the morning of postoperative day (POD) 1 if the drainage volume was < 200 mL. If a patient vomited persistently or the drainage volume was > 200 mL, an NGT was maintained. Conventionally, a solid diet was initiated on POD 3. However, this schedule was changed according to clinical observations, such as abdominal swelling, little peristaltic sounds, or vomiting. Drainage fluid biochemistry was measured for the presence of postoperative bile leakage or POPF by countering bilirubin and amylase on PODs 1, 3, 5, and 7. Drains were removed if there were no signs of postoperative biliary leakage, POPF, or chylorrhea. Postoperatively, a proton pump inhibitor was administered orally from POD 1 to prevent gastrointestinal ulceration. Octreotide administration was determined according to the attending surgeon's preferences (from POD 0 to the day of drainage tubes removing). Pancreatic enzyme supplementation, mosapride citrate hydrate, or Japanese herbal medicine daikenchuto was administered according to the patient's condition.

### Postoperative complication definition

#### DGE

DGE was defined and graded according to the International Study Group of Pancreatic Surgery consensus definition published in 2007 as follows: Grade A, NGT insertion after POD 3 or the inability to tolerate intake of solid diet by POD 7; Grade B, NGT required for 8–14 days postoperatively, NGT reinsertion after POD 7, or the inability to tolerate solid diet by POD 14; and Grade C, NGT required for > 14 days postoperatively, NGT reinsertion after POD 14, or the inability to tolerate solid diet by POD 21 [4].

#### POPF

According to the 2016 update of the International Study Group for Pancreatic Fistula definition and grading of POPF, POPF is defined as a drain output amylase level > 3 times the upper limit of the institutional normal serum amylase activity. Grade A POPF is defined and termed as a "biochemical leak", because it has no clinical importance and is no longer referred to as a true pancreatic fistula. Grade B requires a change in postoperative management; drains are either left in place for > 3 weeks or repositioned through endoscopic or percutaneous procedures. Grade C refers to POPFs that require reoperation or lead to single or multiple organ failure and/or mortality attributable to pancreatic fistula [14].

#### Other complications

Postoperative intra-abdominal infection was defined as the verification of a positive bacterial culture from intra-abdominal drainage tubes after surgery. Sepsis was defined as the detection of bacteria and/or fungi in blood cultures.

### Running pattern of LGV classification

Running pattern of LGV was detected by using computed tomography performed before and after surgery and intraoperative findings from operation records. We performed computed tomography on seven days from surgery routinely. Then, we have investigated that the LGV was preserved or not in postoperative computed tomography. Moreover, we have added presence of LGV ligation in operation record. We investigated the anatomy of the LGV and classified it into four types according to the running pattern of the LGV, ventral or distal route of the common hepatic artery (CHA) and splenic artery (SpA) arcade, and joining point to the veins of the portal vein (PV) or splenic vein (SpV). Previous reports have identified three positions for LGV confluence into major veins: the PV, SpV, and junction of the PV and SpV [15, 16]. However, Kawasaki et al. described a relationship between the LGV and arteries around the pancreas [17]. In this study, we classified the running pattern of the LGV to simplify the classification from a surgical and procedural viewpoint.

**Fig. 1:**
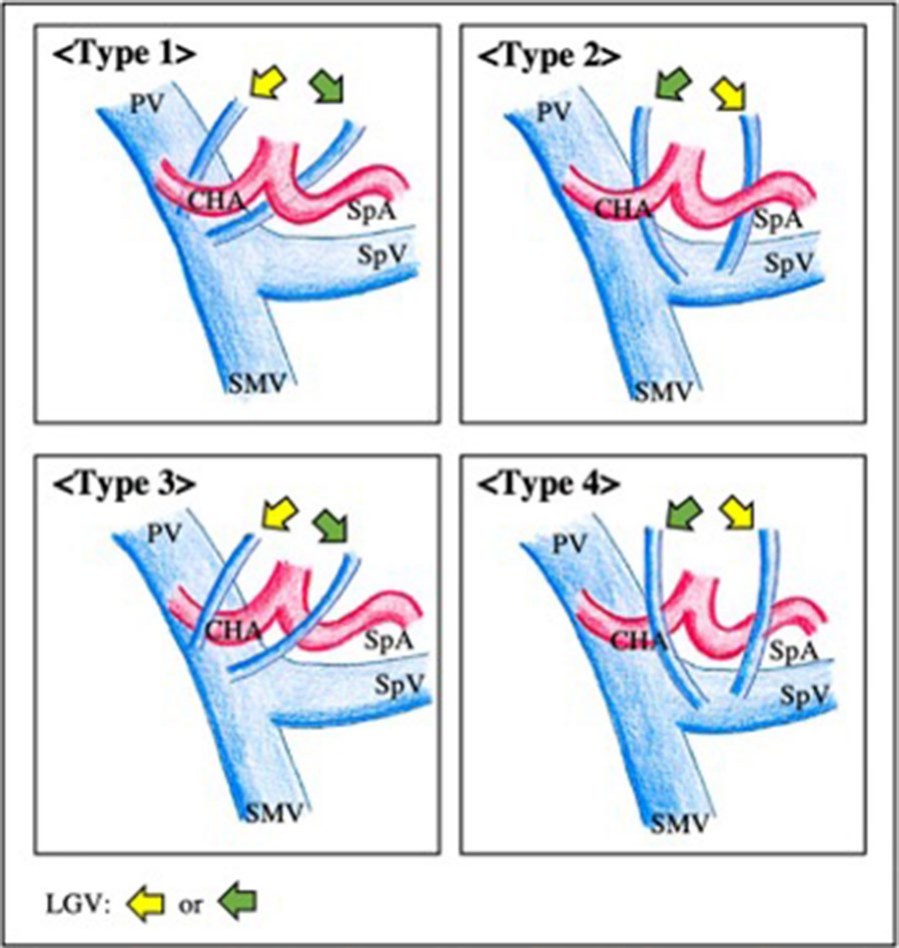
Four running patterns of the left gastric vein. Yellow and green arrowheads point to the left gastric veins. The running patterns indicated by the yellow arrowheads were more frequently ligated than the patterns indicated with green arrowheads. LGV, left gastric vein; CHA, common hepatic artery; SpA, splenic artery; DGA, duodenal gastric artery; PHA, proper hepatic artery; PV, portal vein; SpV, splenic vein; SMV, superior mesenteric vein

Type 1 LGV runs dorsal to the CHA or SpA (mostly dorsal to the CHA) and join the PV. Type 2 LGV runs dorsal to the CHA or SpA (mostly dorsal to the SpA) and joins the SpV. Type 3 LGV runs ventral to the CHA or SpA (mostly ventral of CHA) and joins the PV. Finally, type 4 LGV runs ventral to the CHA or SpA (mostly ventral to the SpA) and joins the SpV (Fig. 1). LGV ligation data were collected from operation records.

We found two cases which LGV flows into the left branch of the portal vein. They were excluded in this study.

### Statistical analyses

All values are expressed as mean and standard deviation. Categorical variables were compared using χ^2^ tests. Statistical significance was set at *p* < 0.05. A logistic regression analysis was used for the multivariate analysis of categorical variables according to risk factors of DGE. All statistical analyses were performed using the JMP 16 software (SAS Institute Japan, Tokyo, Japan).

## Results

### Patient characteristics

A total of 105 patients underwent SSPPD for pancreatic head tumours, periampullary tumours, or diseases manifesting as pancreatic masses between January 2016 and September 2021. The patient characteristics are described in Additional File 1. The most common diagnosis was pancreatic adenocarcinoma (54.3%).

### Frequency of running pattern of LGV and LGV ligation

We investigated the running pattern of the LGV in all the patients and found that 47 cases (44.8%) were type 1, 23 (21.9%) were type 2, 12 (11.4%) were type 3, and 23 (21.9%) were type 4. We also examined which type of running pattern of the LGV was most frequently ligated. The number of ligated LGVs according to type was as follows: type 1, six cases (12.8%); type 2, one case (4.4%); type 3, nine cases (75.0%); and type 4, three cases (13.0%) (Table 1).

**Table 1:** Running pattern of LGV frequency and association between running patterns of the left gastric vein and ligation rate

Running pattern of LGV	Patients (%)	Ligation (%)
Type 1	47 (44.8)	6 (12.8)
Type 2	23 (21.9)	1 (4.4)
Type 3	12 (11.4)	9 (75.0)
Type 4	23 (21.9)	3 (13.0)

### Intraoperative findings

Intraoperative findings are shown in Table 2. In this study, all surgical procedures were performed using the SSPPD. Reconstruction techniques (modified Child method), reconstruction method (Roux-en-Y or Billroth II), and alimentary tract reconstruction method (automatic stapling device or hand-sewn anastomosis) were determined according to the operating surgeon's preferences. In 19 patients (18.1%), the LGV was ligated during surgery.

**Table 2:** Intraoperative findings

Factors	Patients (n = 105)
Procedure (%)	
Subtotal stomach-preserving pancreaticoduodenectomy	105 (100.0)
Pancreatic gland texture (%)	
Hard	44 (41.9)
Soft	61 (58.1)
Pancreas duct size (mm), mean ± standard deviation	4.1 ± 2.2
Reconstruction technique (%)	
Modified Child method	105 (100.0)
Reconstruction method (%)	
Roux-en-Y reconstruction	50 (47.6)
Billroth II reconstruction	55 (52.4)
Route for alimentary tract reconstruction (%)	
Antecolic route	105 (100.0)
Alimentary tract reconstruction method (%)	
Automatic stapling device	95 (90.5)
Hand-sewn anastomosis	10 (9.5)
Vascular resection (%)	
Portal vein	20 (19.0)
Portal vein + proper hepatic artery	1 (1.0)
Portal vein + right hepatic artery	1 (1.0)
None	83 (79.0)
Blood loss (g), mean ± standard deviation	479.5 ± 383.0
Intraoperative transfusion (yes, %)	12 (11.4)
Operative time (min), mean ± standard deviation	527 ± 106
Joint point of LGV (%)	
Portal vein	58 (55.2)
Splenic vein	47 (44.8)
Running of LGV (%)	
Retroarterial route	70 (66.7)
Antearterial route	35 (33.3)
LGV ligation (Yes, %)	19 (18.1)

### Surgical outcomes

Postoperative factors are shown in Table 3. The frequency of all DGE grades after surgery was 20.0%. The frequency of grade B or C pancreatic fistulas was 33.3%. Wound infection occurred in 9.5% of patients and intra-abdominal infection developed in 22.9% of patients after surgery. Only one case of mortality occurred and was caused by disseminated intravascular coagulation after surgery. The deceased patient's right hepatic artery had to be resected and reconstructed because of cancer invasion. The reconstructed right hepatic artery did not develop occlusion but indicated stenosis, which led to the development of multiple liver abscesses and disseminated intravascular coagulation. The patient's general condition worsened, and he was unable to tolerate surgical or non-surgical treatment.

**Table 3:** Postoperative factors

Factors	Patients (n = 105)
Delayed gastric emptying (%)	
None	84 (80.0)
Grade A	14 (13.3)
Grade B	5 (4.8)
Grade C	2 (1.9)
Pancreatic fistula (%)	
None	54 (51.4)
Grade A	16 (15.2)
Grade B	33 (31.4)
Grade C	2 (1.9)
Wound infection (yes, %)	10 (9.5)
Intra-abdominal infection (yes, %)	24 (22.9)
Sepsis (yes, %)	12 (11.4)
> 30 days mortality (yes, %)	1 (1.0)
Postoperative hospitalisation (days), mean ± standard deviation	24.2 ± 14.5

### Univariate and multivariate analysis between DGE and non-DGE cases

Thereafter, we investigated the risk factors for DGE after surgery. The prevalence of preoperative obstructive jaundice was significantly higher in the DGE group than in the non-DGE group (66.7% vs. 40.5%, *p* = 0.0306). Only LGV ligation showed a significant difference between the DGE and non-DGE groups (57.1% vs. 8.3%, *p* < 0.0001) in intraoperative factors. On comparing the DGE and non-DGE groups, grade B or C pancreatic fistula (57.1% vs. 27.4%, *p* = 0.0116) and sepsis (28.6% vs. 7.1%, *p* = 0.0123) emerged as risk factors for the development of DGE after surgery. Postoperative hospitalisation was significantly longer in the DGE group than in the non-DGE group.

We subjected the risk factors for DGE revealed in the univariate analysis to a multivariate analysis and found that LGV ligation was the only independent risk factor for DGE after SSPPD (odds ratio: 13.60, 95% CI: 3.80–48.68, *p* < 0.0001) (Table 4).

**Table 4:** Univariate and multivariate analysis of the association between delayed gastric emptying and operative factors

Factors	Univariate			Multivariate	
	DGE				
	Yes (n = 21)	No (n = 84)	*P*-value	Odds ratio (95% CI)	*P*-value
Sex (male, %)	13 (61.9)	51 (60.7)	0.9203		
Age (years), mean ± standard deviation	71 ± 8	70 ± 10	0.7734		
BMI (kg/m^2^), mean ± standard deviation	22.1 ± 2.9	20.9 ± 3.6	0.1746		
Diabetes mellitus (yes, %)	5 (23.8)	33 (39.3)	0.1759		
Current smoker (yes, %)	2 (9.5)	14 (16.7)	0.3933		
Preoperative obstructive jaundice (Yes, %)	14 (66.7)	34 (40.5)	0.0306	3.13 (0.91–10.82)	0.0710
Tumor size (mm), mean ± standard deviation	24.8 ± 2.6	26.2 ± 1.3	0.6037		
Blood vessels invasion (yes, %)	3 (14.9)	19 (22.6)	0.3848		
Malignancy (yes, %)	18 (85.7)	69 (82.1)	0.6925		
Pancreatic gland texture (hard, %)	7 (33.3)	37 (44.1)	0.3689		
Portal vein resection (yes, %)	3 (14.3)	16 (19.1)	0.7895		
Blood loss (g), mean ± standard deviation	368 ± 243	507 ± 407	0.1354		
Intraoperative transfusion (yes, %)	2 (9.5)	10 (11.9)	0.6383		
Operative time (min), mean ± standard deviation	498 ± 109	534 ± 104	0.1574		
Running pattern of LGV			0.0011		
Type 1	11 (52.4)	36 (42.9)			
Type 2	0 (0.0)	23 (27.4)			
Type 3	6 (28.6)	6 (7.1)			
Type 4	4 (19.1)	19 (22.6)			
LGV ligation (yes, %)	12 (57.1)	7 (8.3)	< 0.0001	13.60 (3.80–48.68)	< 0.0001
Stapling device for gastroenterostomy (yes, %)	18 (85.7)	77 (91.7)	0.4273		
Roux-en-Y reconstruction (yes, %)	8 (38.1)	47 (56.0)	0.1428		
Grade B or C pancreatic fistula (yes, %)	12 (57.1)	23 (27.4)	0.0116	1.70 (0.32–9.17)	0.5357
Wound infection (yes, %)	4 (19.1)	6 (7.1)	0.1242		
Intra-abdominal infection (yes, %)	8 (38.1)	16 (19.1)	0.0749		
Sepsis (yes, %)	6 (28.6)	6 (7.1)	0.0123	1.75 (0.31–9.80)	0.5231
Postoperative hospitalisation (days), mean ± standard deviation	33 ± 12	22 ± 14	0.0017		

### Univariate and multivariate analysis between DGE and non-DGE cases in LGV ligation group

Moreover, we also investigated the risk factors for DGE after surgery in LGV ligation group. On comparing the DGE and non-DGE groups, intra-abdominal infection (83.3% vs. 16.7%, *p* = 0.0051) and sepsis (41.7% vs. 0.0%, *p* = 0.0258) emerged as risk factors for the development of DGE after surgery in LGV ligation group. Postoperative hospitalisation was significantly longer in the DGE group than in the non-DGE group in LGV ligation group.

We subjected the risk factors for DGE in LGV ligation group revealed in the univariate analysis to a multivariate analysis and found that intra-abdominal infection was the only independent risk factor for DGE after SSPPD in LGV ligation group (odds ratio: 12.5, 95% CI: 0.84–186.3, *p* = 0.0413) (Table 5).

**Table 5:** Univariate and multivariate analysis of the association between delayed gastric emptying and operative factors in LGV ligation group

Factors	Univariate			Multivariate	
	DGE				
	Yes (n = 12)	No (n = 6)	*P*-value	Odds ratio (95% CI)	*P*-value
Sex (male, %)	10 (83.3)	3 (50.0)	0.1436		
Age (years), mean ± standard deviation	71 ± 3	69 ± 4	0.6748		
BMI (kg/m^2^), mean ± standard deviation	22.0 ± 1.0	21.9 ± 1.4	0.9534		
Diabetes mellitus (yes, %)	1 (8.3)	2 (33.3)	0.1926		
Current smoker (yes, %)	1 (8.3)	0 (0.0)	0.3594		
Preoperative obstructive jaundice (Yes, %)	8 (66.7)	2 (33.3)	0.1778		
Tumor size (mm), mean ± standard deviation	27.2 ± 3.0	22.3 ± 3.9	0.3391		
Blood vessels invasion (yes, %)	0 (0.0)	0 (0.0)			
Malignancy (yes, %)	11 (91.7)	4 (66.7)	0.0777		
Pancreatic gland texture (hard, %)	2 (16.7)	2 (33.3)	0.4319		
Portal vein resection (yes, %)	0 (0.0)	0 (0.0)			
Blood loss (g), mean ± standard deviation	335 ± 250	524 ± 463	0.2705		
Intraoperative transfusion (yes, %)	1 (8.3)	1 (16.7)	0.6052		
Operative time (min), mean ± standard deviation	476 ± 128	523 ± 60	0.4096		
Running pattern of LGV			0.2284		
Type 1	5 (41.7)	1 (16.7)			
Type 2	0 (0.0)	1 (16.7)			
Type 3	6 (50.0)	3 (50.0)			
Type 4	1 (8.3)	2 (33.3)			
Stapling device for gastroenterostomy (yes, %)	9 (75.0)	6 (100.0)	0.0988		
Roux-en-Y reconstruction (yes, %)	7 (58.3)	1 (16.7)	0.0821		
Grade B or C pancreatic fistula (yes, %)	9 (75.0)	1 (16.7)	0.0861		
Wound infection (yes, %)	4 (33.3)	0 (0.0)	0.0515		
Intra-abdominal infection (yes, %)	10 (83.3)	1 (16.7)	0.0051	12.5 (0.84–186.3)	0.0413
Sepsis (yes, %)	5 (41.7)	0 (0.0)	0.0258	1.69 (0.35–12.8)	0.2551
Postoperative hospitalisation (days), mean ± standard deviation	36 ± 11	16 ± 7	0.0009		

### DGE development with or without LGV ligation in the absence of grade B or C pancreatic fistula group

Furthermore, we investigated DGE development after surgery with or without LGV ligation in the absence of grade B or C pancreatic fistula. DGE was significantly more frequent in the LGV ligation group than in the LGV preservation group (33.3% vs. 10.3%, *p* = 0.0493).

## Discussion

**Fig. 2:**
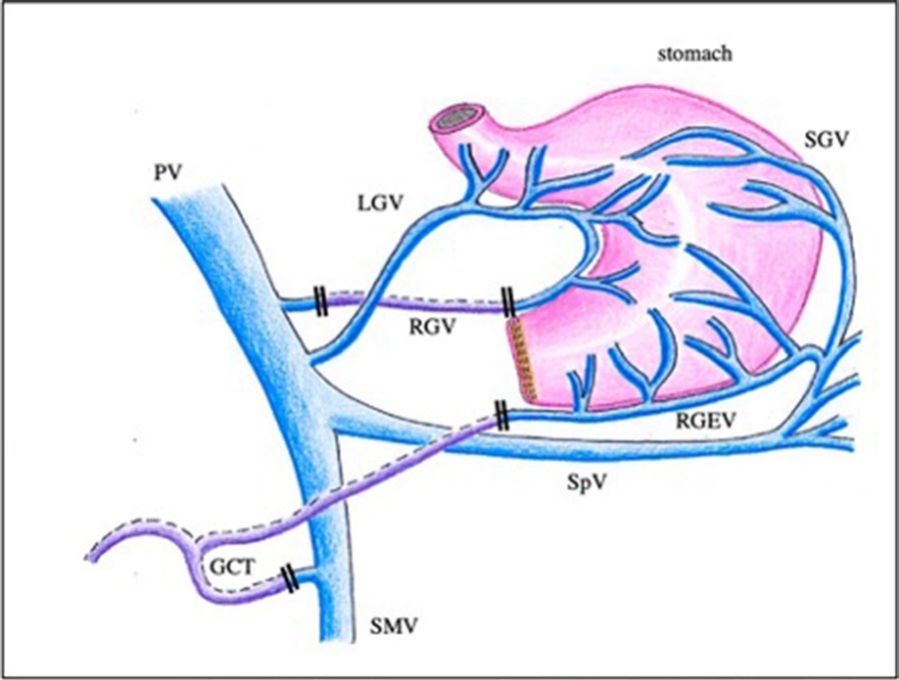
Mechanism of reducing the development of delayed gastric emptying after subtotal stomach-preserving pancreatoduodenectomy by preserving the left gastric vein as suggested. Double black lines indicate the ligation point. PV, portal vein; SpV, splenic vein; SMV, superior mesenteric vein; GCT, gastrocolic trunk; LGV, left gastric vein; RGV, right gastric vein; SGV, short gastric vein; RGEV, right gastroepiploic vein

DGE is a major complication of PD; however, the development and risk factors of DGE are still controversial [1, 4, 5, 7–9]. Physiologically, the causes of DGE after PD are hypothesised as follows: decreased plasma levels of the hormone motilin due to resection of the duodenum and its M-cells [18, 19], damage to branches of the vagus nerve in a dissection of lymph nodes, and ischaemia of the point of the alimentary tract reconstruction [4]. DGE in the postoperative period decreases the quality of life of patients and prolongs hospitalisation by extension of inanition states, ultimately leading to increased health care costs [5]. In this study, DGE after SSPPD was more frequent in the LGV ligation group than in the LGV preservation group. We resected the right gastric artery and vein for routine dissection of #5 lymph nodes in performing SSPPD. LGV ligation might cause stasis of the lesser curvature of the stomach and lead to the development of DGE after SSPPD. Thus, LGV preservation may reduce the rate of DGE development after SSPPD (Fig. 2).

Moreover, LGVs with type 3 running pattern were the most frequently ligated LGV type. A type 3 LGV running ventral to the CHA or SpA and directly joining the PV would obstruct the surgical field of view in the dissection of #12p and/or #8a lymph nodes around the PV and CHA and could be ligated during lymph node dissection. Contrastingly, LGVs with type 1, type 2, and type 4 running patterns remain distant from the lymph node dissection field in SSPPD and thus are less likely to be ligated intraoperatively. It would be essential to preserve the LGV by taping it if it is recognised during surgery.

Several reports have revealed that intra- and postoperative management techniques reduce the incidence of DGE after surgery. Matsumoto et al. reported that the occurrence rate of DGE after surgery was higher in the PPPD group than in the SSPPD group [20]. Postoperative intra-abdominal infection and POPF are well-known risk factors for DGE after PD [7]. Tani et al. first performed a randomised controlled trial to investigate the effect of alimentary tract reconstruction (antecolic or retrocolic route) on DGE after PD [21]. Their results revealed a significantly higher occurrence rate of DGE after PPPD with the retrocolic alimentary route than with the antecolic route. In addition, Toyama et al. failed to prove the noninferiority of retrocolic alimentary tract reconstruction to antecolic alimentary tract reconstruction with respect to the postoperative incidence of DGE [1]. Moreover, Hwang et al. suggested that Braun anastomosis reduced DGE after PD [22], and Shimoda et al. reported that the incidence of DGE after SSPPD can be decreased using Billroth II rather than Roux-en-Y reconstruction for gastrojejunostomy [9].

First, all cases in this study were SSPPD; therefore, the effect of PPPD on the development of DGE after surgery could not be determined. We propose that the mechanism of DGE development between SSPPDs and PPPDs should be dissociated with or without resection of the pyloric ring. In our study, there was no significant difference in the data between patients with and without intra-abdominal infection. We also examined the effect of LGV ligation for DGE after SSPPD in patients without grade B or C POPF. The LGV ligation group showed a significantly higher incidence of DGE after SSPPD than the LGV preservation group. This result suggests that LGV ligation as a risk factor for DGE after SSPPD is unrelated to the presence of POPF. The effect of the alimentary tract reconstruction route (antecolic or retrocolic route) on DGE was also unclear in this study because antecolic route reconstruction was performed in all cases. Braun anastomosis was routinely performed in all Billroth II reconstruction cases. Moreover, we investigated the effect of LGV ligation on DGE after SSPPD in patients who underwent Roux-en-Y reconstruction. The LGV ligation group showed a significantly higher rate of DGE incidence after SSPPD than the LGV preservation group (45.5% vs. 6.8%, *p* = 0.0012). We also examined the effect of LGV ligation on DGE after surgery in patients who underwent Billroth II reconstruction. The LGV ligation group showed a significantly higher rate of DGE incidence after SSPPD than the LGV preservation group (87.5% vs. 14.3%, *p* = 0.0012). These findings revealed that LGV ligation was a risk factor for DGE after SSPPD but was unrelated to the reconstruction methods.

Regarding the possibility of damage to the vagus nerve in DGE after SSPPD, Xu et al. suggested that preservation of the hepatic branches of the vagus nerve reduces the development of DGE after laparoscopic PD [23]. Nevertheless, the LGV runs closer to the caudal side of the hepatic branch of the vagus nerve, indicating that LGV ligation might be related to the damage of the left gastric artery plexus from the posterior trunk of the vagus nerve anatomically [24]. LGVs running ventral to the artery are easily ligated during lymph node dissection. Therefore, we suggest the possibility of injury to the left gastric artery plexus during LGV ligation. DGE is also a complication of distal gastrectomy [25]. Kim et al. demonstrated that the preservation of the proximal side of the bifurcation of the hepatic branch from the anterior trunk of the vagus nerve and celiac branch from the posterior trunk of the vagus nerve improves gastric function after distal gastrectomy [26]. We were unable to corroborate this observation in our study because we did not explore the damage to the vagus nerve branches; nevertheless, more popularisation of the laparoscopic PD procedure would enable us to visualise and investigate the running patterns of the thin vagus nerve branches in more detail.

This study has some limitations. First, we selected patients from a single centre. A multicentre study with a larger number of patients and greater variation in surgical techniques and surgeons would help us reach more definitive conclusions. Second, this was a retrospective study, and may have been subject to investigative bias. Further randomised controlled multicentre studies are needed.

## Conclusions

We investigated variations in the running pattern of LGV and effect of LGV ligation on the occurrence rate of DGE after SSPPD. We found that type 3 LGVs running ventral to the CHA or SpA and joining the PV were most frequently ligated, and LGV ligation was an independent risk factor for DGE after SSPPD. Type 3 LGVs impede lymph node dissection around the PV and/or CHA and must be ligated. If the LGV is recognised during surgery, it should be preserved, as far as possible, by taping with vessel tapes. Since preservation of the LGV maintains gastric drainage venous flow, we suggest that the LGV should be preserved as far as possible to reduce gastric stasis and prevent the development of DGE after SSPPD.

## Supplementary Information

**Additional file 1**. Patient characteristics.

## Data Availability

The datasets used and/or analysed during the current study are available from the corresponding author on reasonable request.

## Abbreviations

LGV: Left gastric vein
SSPPD: Subtotal stomach-preserving pancreatoduodenectomy
DGE: Delayed gastric emptying
CHA: Common hepatic artery
SpA: Splenic artery
PV: Portal vein
PD: Pancreatoduodenectomy
POPF: Postoperative pancreatic fistula
PPPD: Pylorus-preserving pancreatoduodenectomy
POD: Postoperative day
NGT: Nasogastric tube
SpV: Splenic vein

## References

[1] Toyama H, Matsumoto I, Mizumoto T, Fujita H, Tsuchida S, Kanbara Y (2021). Influence of the retrocolic versus antecolic route for alimentary tract reconstruction on delayed gastric emptying after pancreatoduodenectomy: a multicenter, noninferiority randomized controlled trial. Ann Surg.

[2] Cameron JL, He J (2015). Two thousand consecutive pancreaticoduodenectomies. J Am Coll Surg.

[3] Nishizaki T, Ijichi H, Terashi T, Shimabukuro R, Maruyama S, Guntani A (2009). Pancreatogastrostomy with an elastic purse-string suture around the gastric orifice after pancreatoduodenectomy. Surg Today.

[4] Wente MN, Bassi C, Dervenis C, Fingerhut A, Gouma DJ, Izbicki JR (2007). Delayed gastric emptying (DGE) after pancreatic surgery: a suggested definition by the International Study Group of Pancreatic Surgery (ISGPS). Surgery.

[5] Werba G, Sparks AD, Lin PP, Johnson LB, Vaziri K (2022). The PrEDICT-DGE score as a simple preoperative screening tool identifies patients at increased risk for delayed gastric emptying after pancreaticoduodenectomy. HPB (Oxford).

[6] Jimenez RE, Fernandez-del Castillo C, Rattner DW, Chang Y, Warshaw AL (2000). Outcome of pancreaticoduodenectomy with pylorus preservation or with antrectomy in the treatment of chronic pancreatitis. Ann Surg.

[7] Ellis RJ, Gupta AR, Hewitt DB, Merkow RP, Cohen ME, Ko CY (2019). Risk factors for post-pancreaticoduodenectomy delayed gastric emptying in the absence of pancreatic fistula or intra-abdominal infection. J Surg Oncol.

[8] Hochwald SN, Grobmyer SR, Hemming AW, Curran E, Bloom DA, Delano M (2010). Braun enteroenterostomy is associated with reduced delayed gastric emptying and early resumption of oral feeding following pancreaticoduodenectomy. J Surg Oncol.

[9] Shimoda M, Kubota K, Katoh M, Kita J (2013). Effect of Billroth II or Roux-en-Y reconstruction for the gastrojejunostomy on delayed gastric emptying after pancreaticoduodenectomy: a randomized controlled study. Ann Surg.

[10] Kurosaki I, Hatakeyama K (2005). Preservation of the left gastric vein in delayed gastric emptying after pylorus-preserving pancreaticoduodenectomy. J Gastrointest Surg.

[11] Japan Pancreas Society (2017). Classification of pancreatic carcinoma.

[12] Fujii T, Sugimoto H, Yamada S, Kanda M, Suenaga M, Takami H (2014). Modified Blumgart anastomosis for pancreaticojejunostomy: technical improvement in matched historical control study. J Gastrointest Surg.

[13] Kakita A, Takahashi T, Yoshida M, Furuta K (1996). A simpler and more reliable technique of pancreatojejunal anastomosis. Surg Today.

[14] Bassi C, Marchegiani G, Dervenis C, Sarr M, Abu Hilal M, Adham M (2017). The 2016 update of the International Study Group (ISGPS) definition and grading of postoperative pancreatic fistula: 11 years after. Surgery.

[15] Sakaguchi T, Suzuki S, Morita Y, Oishi K, Suzuki A, Fukumoto K (2010). Analysis of anatomic variants of mesenteric veins by 3-dimensional portography using multidetector-row computed tomography. Am J Surg.

[16] Nishino H, Zimmitti G, Ohtsuka T, Abu Hilal M, Goh BK, Kooby DA (2022). Precision vascular anatomy for minimally invasive distal pancreatectomy: a systematic review. J Hepatobil Pancreat Sci.

[17] Kawasaki K, Kanaji S, Kobayashi I, Fujita T, Kominami H, Ueno K (2010). Multidetector computed tomography for preoperative identification of left gastric vein location in patients with gastric cancer. Gastric Cancer.

[18] Tack J, Deloose E, Ang D, Scarpellini E, Vanuytsel T, Van Oudenhove L (2016). Motilin-induced gastric contractions signal hunger in man. Gut.

[19] Tanaka M, Sarr MG (1988). Role of the duodenum in the control of canine gastrointestinal motility. Gastroenterology.

[20] Matsumoto I, Shinzeki M, Asari S, Goto T, Shirakawa S, Ajiki T (2014). A prospective randomized comparison between pylorus- and subtotal stomach-preserving pancreatoduodenectomy on postoperative delayed gastric emptying occurrence and long-term nutritional status. J Surg Oncol.

[21] Tani M, Terasawa H, Kawai M, Ina S, Hirono S, Uchiyama K (2006). Improvement of delayed gastric emptying in pylorus-preserving pancreaticoduodenectomy: results of a prospective, randomized, controlled trial. Ann Surg.

[22] Hwang HK, Lee SH, Han DH, Choi SH, Kang CM, Lee WJ (2016). Impact of Braun anastomosis on reducing delayed gastric emptying following pancreaticoduodenectomy: a prospective, randomized controlled trial. J Hepatobiliary Pancreat Sci.

[23] Li X, Qin T, Zhu F, Wang M, Dang C, He L (2021). Clinical efficacy of the preservation of the hepatic branch of the vagus nerve on delayed gastric emptying after laparoscopic pancreaticoduodenectomy. J Gastrointest Surg.

[24] Sişu AM, Stana LG, Petrescu CI, Motoc A (2012). Macroscopic, mesoscopic and microscopic morphology of the gastric plexus–ontogeny of the celiac ganglion. Rom J Morphol Embryol.

[25] Tomita R, Fujisaki S, Tanjoh K, Fukuzawa M (2000). Relationship between gastroduodenal interdigestive migrating motor complex and quality of life in patients with distal subtotal gastrectomy for early gastric cancer. Int Surg.

[26] Kim SM, Cho J, Kang D, Oh SJ, Kim AR, Sohn TS (2016). A randomized controlled trial of vagus nerve-preserving distal gastrectomy versus conventional distal gastrectomy for postoperative quality of life in early stage gastric cancer patients. Ann Surg.

