# Supplementary material for: Ligation of left gastric vein may cause delayed gastric emptying after pancreatoduodenectomy: a retrospective study
Source: BMC Gastroenterol. 2022 Aug 26;22:398. doi: 10.1186/s12876-022-02478-5 (PMC9414412; doi:10.1186/s12876-022-02478-5)
Supplement: Supplementary file 1 — Additional file 1. Patient characteristics. [file 12876_2022_2478_MOESM1_ESM.docx]

**Additional File 1.** Patients’ characteristics

| **Factors** | **Patients (n=105)** | | |
| --- | --- | --- | --- |
| Age (years), mean ± standard deviation | 70 ± 9 | | |
| Sex (male, %) | 64 (61.0) | | |
| BMI (kg/m^2^), mean ± standard deviation | 21.1 ± 3.5 | | |
| ASA classification (%) |  | | |
| 1 | 10 (9.5) | | |
| 2 | 80 (76.2) | | |
| 3 | 15 (14.3) | | |
| Diabetes mellitus (yes, %) | 38 (36.2) | | |
| Current smoker (yes, %) | 16 (15.2) | | |
| >10% weight loss in past 6 months (yes, %) | 9 (8.6) | | |
| Preoperative obstructive jaundice (yes, %) | 48 (45.7) | | |
| Preoperative biliary stent replacement (yes, %) | 47 (44.8) | | |
| Tumor size (mm) , mean ± standard deviation | 26.0 ± 11.5 | | |
| Blood vessels invasion (yes, %) | 22 (21.0) | | |
| Malignancy (yes, %) | 87 (82.9) | | |
| Diagnosis (%) |  | | |
| Pancreatic adenocarcinoma | | 57 (54.3) |  |
| Distal cholangiocarcinoma | | 23 (21.9) |  |
| Intraductal papillary mucinous neoplasm | | 9 (8.6) |  |
| Pancreatic neuroendocrine tumour | | 4 (3.8) |  |
| Intraductal papillary mucinous carcinoma | | 3 (2.9) |  |
| Duodenal carcinoma | | 2 (1.9) |  |
| Solid pseudopapillary neoplasm | | 2 (1.9) |  |
| Papillary neuroendocrine carcinoma | | 1 (1.0) |  |
| Renal carcinoma pancreatic metastasis | | 1 (1.0) |  |
| Mucinous cystic neoplasm | | 1 (1.0) |  |
| Serous cystic neoplasm | | 1 (1.0) |  |
| Biliary intraepithelial neoplasia | | 1 (1.0) |  |
